# Loss of MeCP2 Causes Urological Dysfunction and Contributes to Death by Kidney Failure in Mouse Models of Rett Syndrome

**DOI:** 10.1371/journal.pone.0165550

**Published:** 2016-11-09

**Authors:** Christopher S. Ward, Teng-Wei Huang, José A. Herrera, Rodney C. Samaco, Meagan R. Pitcher, Alan Herron, Steven A. Skinner, Walter E. Kaufmann, Daniel G. Glaze, Alan K. Percy, Jeffrey L. Neul

**Affiliations:** 1 Department of Pediatrics, Baylor College of Medicine, Houston, TX 77030, United States of America; 2 Department of Molecular and Human Genetics, Baylor College of Medicine, Houston, TX 77030, United States of America; 3 Program in Developmental Biology, Baylor College of Medicine, Houston, TX 77030, United States of America; 4 Program in Translational Biology and Molecular Medicine, Baylor College of Medicine, Houston, TX 77030, United States of America; 5 Center for Comparative Medicine, Baylor College of Medicine, Houston, TX 77030, United States of America; 6 Greenwood Genetic Center, Greenwood, SC 29646, United States of America; 7 Boston Children’s Hospital, Boston, MA 02115, United States of America; 8 University of Alabama, Birmingham, Birmingham, AL 35294, United States of America; 9 Jan and Dan Duncan Neurological Research Institute, Texas Children’s Hospital, Houston, TX 77030, United States of America; Universita degli Studi dell'Insubria, ITALY

## Abstract

Rett Syndrome (RTT) is a neurodevelopmental disorder characterized by loss of acquired skills during development, autonomic dysfunction, and an increased risk for premature lethality. Clinical experience identified a subset of individuals with RTT that present with urological dysfunction including individuals with frequent urinary tract infections, kidney stones, and urine retention requiring frequent catheterization for bladder voiding. To determine if urologic dysfunction is a feature of RTT, we queried the Rett Syndrome Natural History Study, a repository of clinical data from over 1000 individuals with RTT and found multiple instances of urological dysfunction. We then evaluated urological function in a mouse model of RTT and found an abnormal pattern of micturition. Both male and female mice possessing *Mecp2* mutations show a decrease in urine output per micturition event. Furthermore, we identified signs of kidney failure secondary to urethral obstruction. Although genetic strain background significantly affects both survival and penetrance of the urethral obstruction phenotype, survival and penetrance of urethral obstruction do not directly correlate. We have identified an additional phenotype caused by loss of MeCP2, urological dysfunction. Furthermore, we urge caution in the interpretation of survival data as an endpoint in preclinical studies, especially where causes of mortality are poorly characterized.

## Introduction

Rett Syndrome (RTT, OMIM 312750) is a neurodevelopmental disorder that affects approximately 1 in 10^4^ live female births [1, 2]. More than 95% of typical RTT cases are caused by mutations in the X-linked gene *Methyl-CpG-binding protein 2* (*MECP2*, OMIM 300005) [2, 3]. Individuals with RTT show normal early development followed by a period of regression during which acquired spoken language ability and fine motor function are lost, gait is compromised, and purposeful hand skills are replaced by stereotypic hand movements such as hand wringing [4].

There are several additional features that are commonly associated with RTT including disrupted sleep, cold extremities, cardiac function abnormalities, and breathing disturbances including bouts of hyperventilation and apnea [4–6]. It is also possible for males to be born with similar disease causing *MECP2* mutation; however, they are typically classified as having severe congenital encephalopathy with more severe manifestations of autonomic disturbances leading to lethality within the first few years of life [7]. These MeCP2 deficit-dependent symptoms indicate that the autonomic nervous system is disrupted in many of the individuals with RTT. Autonomic disturbances have also been reproduced in multiple mouse models of RTT where the *Mecp2* locus has been modified with null or disease-causing mutations [5, 8–11].

Disruption of the autonomic nervous system may contribute to additional health and quality of life impairments that remain to be identified in human cases of RTT and the animal models of the disease. We observed multiple cases of RTT in the clinic that present with frequent urological comorbidities (JLN, WEK personal observations). The occurrence of urological complications in RTT seemed to be a potential consequence of the disrupted autonomic nervous system function. Indeed, urological problems are present in other neurological disorders including Parkinson’s disease, Huntington chorea, cerebral palsy, Down syndrome, and *MECP2* duplication syndrome [12–16]. We examined a large dataset of human cases to determine if urological complications are a common feature of RTT, and identified several individuals with urological dysfunction. We also examined whether urological abnormalities were also present in a mouse model of RTT, and observed deficits in the patterns of micturition and evidence of urological dysfunction contributing to lethality in male mice lacking MeCP2. These data suggest that urological problems are a result of disrupted MeCP2 function, and further work is advised to understand the exact etiology and frequency of these problems in people with RTT.

## Materials and Methods

### Human data

Human data were collected as part of the Rett Syndrome Natural History Study [17]. Protocols and consents were approved by the Institutional Review Boards of Baylor College of Medicine, University of Alabama-Birmingham, Greenwood Genetic Center, and Boston Children’s Hospital. We obtained written consent from parents or guardians of the individuals with RTT, as the subjects are unable to communicate.

### Animals used in experiments

All research and animal care procedures were approved by the Baylor College of Medicine Institutional Animal Care and Use Committee (protocol AN4972) and housed in the Association for Assessment and Accreditation of Laboratory Animal Care-approved animal facility at Baylor College of Medicine. All efforts were taken to minimize suffering. Humane endpoints were used for survival studies. Animals were euthanized when observed in a moribund state as defined by hypoactivity, sudden weight loss, decreased body temperature, or respiratory distress. Moribund mice were either anesthetized with Avertin (1.25% tribromoethanol/amyl alcohol solution, i.p.) using a dose of 0.02 ml/g for terminal tissue collection described later, or euthanized by CO_2_ inhalation in accordance with recommendations of the American Veterinary Medical Association[18]. Animals were monitored daily by animal husbandry staff or investigators. The timing of the onset of the moribund state and premature lethality is similar to observations made in previous publications utilizing mice lacking MeCP2 expression [19, 20]. Results of necropsy performed on moribund mice are presented in the results.

*Mecp2*^*tm*.*1*.*1Bird*^ mice [20] were obtained as a gift from Dr. Adrian Bird (University of Edinburgh, Edinburgh, UK). This line was maintained on several strain backgrounds by backcrossing to a 129S6 strain background for >10 generations, backcrossing to a FVBHSD strain background for >10 generations as well as continuing to maintain it on C57BL/6J. Mice possessing the *Mecp2*^*tm1*.*1Jae*^ and *Mecp2*^*tm2Bird*^ alleles were obtained from the Mutant Mouse Regional Resource Center and Jackson Labs respectively and maintained on a C57Bl/6J strain background [19, 21]. Isogenic hybrid strains were created by crossing female *Mecp2*^*tm1*.*1Bird/+*^ mice of the different strain backgrounds to male wild-type mice of other strain backgrounds (C57BL/6J, FVBHSD, 129S6). 129B6F1, 129FVBF1, FVB129F1, FVBB6F1, and B6FVBF1 hybrid strains were generated. 129FVBF1 and FVB129F1 results, and FVBB6F1 and B6FVBF1 results were respectively pooled as 129FVBF1 and FVBB6F1 results since no differences were observed in survival and urological phenotypes between the reciprocal hybrid crosses.

### Void stain on paper test

Testing patterns of urine output were performed similarly to methods described by Sugino and colleagues [22]. Briefly, male and female mice were placed on a wire grid and covered by a circular plastic chamber 6.5 inches in diameter. A piece of filter paper was placed under the grid to capture urine. Mice were left undisturbed for 3 hours and provided with water. Micturition patterns and volumes were quantified by imaging the filter paper with UV-light and determining the number and surface area of the spots with ImageJ software [23]. Spot area was converted to liquid volume using the spot sizes of known volumes of 0.04% bromophenol blue in normal saline solution loaded onto filter paper.

### Blood analysis

Male mice were anesthetized with Avertin (1.25% tribromoethanol/amyl alcohol solution, i.p.) using a dose of 0.02 ml/g and then blood was collected via cardiac puncture of the left ventricle. Serum was separated by centrifugation at 4000 x g for 4 minutes, and the supernatant transferred to a new tube. Serum chemistry analysis was performed by the Pathology Core at Baylor College of Medicine using an automated serum chemistry analyzer.

### Histology

Male mice were anesthetized with Avertin as described above and euthanized by cervical dislocation. Tissue samples were obtained and fixed overnight in 10% buffered formalin. Tissue processing, paraffin embedding, sectioning at 5μm thickness, and Hematoxylin and Eosin staining were performed by the Baylor College of Medicine Pathology Core according to their standard protocols.

### Statistical analyses

All statistics were performed using SPSS or Excel on a PC. Data from the Rett Syndrome Natural History Study were analyzed using Mann-Whitney U non-parametric tests for correlation of urologic dysfunction with disease severity, or Chi-Squared tests for correlation of urologic dysfunction with drug usage. Data from mice with *Mecp2* mutations were analyzed by ANOVA to compare effects of genotype. Survival analysis was performed using Kaplan–Meier survival analysis, with Tarone–Ware method applied to detect differences in survival between genotype groups. In the event of multiple pairwise comparisons, p values were adjusted by *post hoc* correction.

## Results

### Urological dysfunction can be found in individuals with RTT

We queried the Rett Syndrome Natural History Study (RSNHS) to determine whether urological dysfunction is present in people with RTT. The RSNHS is a NIH funded longitudinal study of over 1000 individuals with RTT or other *MECP2* related disorders that have undergone thorough genetic testing for *MECP2* mutations and clinical characterization on a yearly or semiannual basis for 8 years. We examined the entries from the “Current History” health questionnaire, which includes questions about recent hospitalizations, surgeries, and co-morbid medical problems, and is updated at each of the study visits. From the 1165 individuals that “Current History” health questionnaire data is available, 2960 SNOMED code entries were identified indicating causes of hospitalizations, surgeries or other co-morbid medical issues. These entries were then manually curated for urological events and classified as renal tubular acidosis (RTA), urinary tract infection (UTI), kidney stones, lithotripsy, nephrectomy, nephrostomy, urine retention, neurogenic bladder, vesicostomy, vesicouretaric reflux, ureteral stent placement, and cystic kidney.

The frequency of the reported conditions is presented, subdivided across the diagnoses of typical RTT, atypical RTT (including the preserved speech, early seizure, and congenital variants of RTT), and other non-RTT cases with *MECP2* related mutations (**Table 1**). The data obtained from the RSNHS confirm the occurrence of urologic complications within a subset of patients with *MECP2* mutations.

**Table 1:** Incidence of urological dysfunction in RTT and individuals with MECP2 mutations.

	Classic		Atypical		Other Non-RTT		Total	
*Total Patient Pool*	905		162		98		1165	
RTA	2	(0.22%)	0	(0.00%)	0	(0.00%)	2	(0.17%)
UTI	39	(4.31%)	5	(3.09%)	3	(3.06%)	47	(4.03%)
Stones	26	(2.87%)	5	(3.09%)	1	(1.02%)	32	(2.75%)
Lithotripsy	5	(0.55%)	2	(1.23%)	0	(0.00%)	7	(0.60%)
Nephrectomy	1	(0.11%)	0	(0.00%)	0	(0.00%)	1	(0.09%)
Nephrostomy	3	(0.33%)	0	(0.00%)	0	(0.00%)	3	(0.26%)
Retention	8	(0.88%)	2	(1.23%)	1	(1.02%)	11	(0.94%)
Neurogenic Bladder	3	(0.33%)	0	(0.00%)	0	(0.00%)	3	(0.26%)
Vesicostomy	2	(0.22%)	0	(0.00%)	0	(0.00%)	2	(0.17%)
VU reflux	6	(0.66%)	3	(1.85%)	0	(0.00%)	9	(0.77%)
Uretal Stent	0	(0.00%)	1	(0.62%)	0	(0.00%)	1	(0.09%)
Cystic Kidney	1	(0.11%)	0	(0.00%)	2	(2.04%)	3	(0.26%)
Any of the Above	73	(8.07%)	13	(8.02%)	7	(7.14%)	93	(7.98%)

Within the patients diagnosed with typical RTT we sought to determine the correlation of several clinical features captured by the RSNHS by the Clinical Severity Score [3] and Motor Behavior Assessment questionnaires [24](**Table 2**). Higher Clinical Severity Score and Motor Behavior Assessment values indicate greater severity of dysfunction. Data for these clinical features was available for 866 individuals. Due to the fact that the RSNHS lacks reliable data on the dates during which urologic symptoms occurred, the individuals were subdivided into affected versus unaffected groups. Multiple measurements attributed to the same individual were averaged so that each individual would have one value for each of the clinical features analyzed. Ambulation, hand use and scoliosis were among the features that showed correlation with urologic dysfunction. The p-values presented are uncorrected for multiple testing correction, but a Bonferroni correction would reach significance at 0.00096, so in general the comparisons with p<0.001 are clearly significant.

**Table 2:** Association of clinical features with urological dysfunction in individuals with typical RTT.

	Unaffected (N = 794)	Affected (N = 72)	*p<0.05
	mean±s.e.m.	mean±s.e.m.	**p≤0.001
**Total Clinical Severity Score**	**22.83±0.25**	**25.58±0.75**	**
Onset of Regression	2.45±0.03	2.50±0.11	n.s.
Ambulation at Exam	2.44±0.07	3.11±0.20	*
Autonomic Symptoms at Exam	0.89±0.02	1.08±0.10	n.s.
Epilepsy/Seizures at Exam	0.91±0.04	1.08±0.14	n.s.
Hand Use	2.04±0.03	2.33±0.10	*
Head Growth	2.11±0.05	2.26±0.17	n.s.
Independent Sitting at Exam	1.06±0.05	1.52±0.18	*
Language at Exam	3.10±0.02	3.17±0.05	n.s.
Nonverbal Communication at Exam	1.89±0.03	1.91±0.08	n.s.
Onset of Stereotypes	2.22±0.03	2.06±0.09	n.s.
Respiratory Dysfunction at Exam	1.36±0.03	1.41±0.10	n.s.
Scoliosis	1.32±0.06	2.10±0.21	**
Somatic Growth	1.05±0.04	1.06±0.15	n.s.
**Grand Total for Motor Behavioral Assessment**	**48.09±0.46**	**54.06±1.28**	**
Aggressive Behabior	0.17±0.02	0.07±0.02	n.s.
Air/Sailva Expulsion	1.19±0.03	1.41±0.10	*
Insensitivity to Pain	1.51±0.03	1.57±0.08	n.s.
Ataxia/Apraxia	3.77±0.02	3.81±0.07	n.s.
Biting of Self/Others	0.17±0.01	0.13±0.04	n.s.
Bradykinesia	0.64±0.04	1.10±0.14	**
Breath Holding	1.12±0.03	1.08±0.09	n.s.
Bruxism	0.90±0.03	0.64±0.09	*
Chewing Difficulties	1.81±0.04	2.22±0.14	*
Chorea Athetosis	0.30±0.02	0.44±0.08	*
Deaf/Does Not Follow Verbal Acts	1.74±0.03	1.98±0.11	*
Does Not Reach for Objects/People	2.02±0.05	2.47±0.16	*
Dystonia	1.12±0.03	1.64±0.11	**
Feeding Difficulties	1.49±0.04	1.84±0.15	*
Hand Clumsiness	2.97±0.04	3.39±0.10	**
Hyperreflexia	0.80±0.04	1.21±0.15	*
Hypertonia/Rigidity	1.22±0.05	1.85±0.16	**
Hyperventilation	0.88±0.03	0.83±0.10	n.s.
Hypomimia	0.49±0.03	0.86±0.11	**
Irritability/Crying/Tantrums	0.35±0.02	0.35±0.07	n.s.
Lack of Sustained Interest	1.43±0.03	1.51±0.09	n.s.
Lack Toilet Training	3.29±0.03	3.35±0.09	n.s.
Masturbation	0.08±0.01	0.09±0.03	n.s.
Motor Skills Regression	2.73±0.03	3.10±0.09	**
Mouthing Hands/Objects	1.04±0.04	0.74±0.10	*
Myoclonus	0.23±0.02	0.45±0.08	**
Oculogyric Movements	0.03±0.01	0.09±0.06	n.s.
Over Active/Over Passive	0.78±0.03	0.82±0.10	n.s.
Poor Eye/Social Contact	1.26±0.03	1.26±0.08	n.s.
Scoliosis	1.17±0.05	1.81±0.17	**
Seizures	1.12±0.04	1.40±0.13	*
Self Mutilating Scratching	0.21±0.02	0.16±0.04	n.s.
Speech Disturbance	3.05±0.02	3.12±0.05	n.s.
Stereotypic Hand Activities	3.39±0.03	3.36±0.08	n.s.
Truncal Rocking/Shifting Weight	0.93±0.03	0.91±0.08	n.s.
Vasomotor Disturbance	1.01±0.03	1.17±0.09	*
Verbal Skills Regression	1.73±0.03	1.81±0.11	n.s.

P values determined by Mann-Whitney U non parametric test.

We also sought to determine if any of the medications used by the patients showed positive or negative correlation with the occurrence of urologic dysfunction (**Table 3**). Of the individuals diagnosed with typical RTT, 733 had information on 453 different medications. 152 medications were taken by at least 5 individuals. Chi-Square statistics were calculated for the association of the 152 medications with occurrence of urologic symptoms. A Benjamini-Hochberg procedure was performed on these results to set a false discovery rate of 0.10. Because the timing of urologic symptoms relative to the dates of medication use could not be determined, we performed an analysis on whether the affected and unaffected individuals were ever on the analyzed medications. Of the medications that showed a significant correlation with urologic symptoms, a number are commonly prescribed to treat urologic symptoms such as citric acid, metronidazole, cranberry, and nitrofurantoin. There is also a correlation with anti-seizure medications such as clobazam, gabapentin, lacosamide, and lorazepam. These drugs could potentially cause urinary retention; however, the relationship is complicated because of the observed correlation with increased seizures and urinary tract symptoms presented in Table 2. Finally, we did observe correlation with medication known to cause urinary retention such as carbidopa/levodopa and dicyclomine, however these medications were present in only a small subset of all people who displayed urinary tract symptoms [25, 26].

**Table 3:** Drugs used by individuals with Classic RTT correlated with urological complications.

	On Drug		Not On Drug		(Affected/Unaffected)			
Drug	Affected	Unaffected	Affected	Unaffected	On Drug	Not On Drug	Relative Risk	p value
baclofen	19	61	51	602	0.24	0.08	3.0	4.7E-06
bisacodyl	7	19	63	644	0.27	0.09	3.0	2.1E-03
calcium	24	109	46	554	0.18	0.08	2.4	2.3E-04
carbidopa	3	3	67	660	0.50	0.09	5.4	7.1E-04
carbidopa / levodopa	6	6	64	657	0.50	0.09	5.6	1.5E-06
citric acid	8	4	62	659	0.67	0.09	7.8	1.1E-11
clobazam	6	18	64	645	0.25	0.09	2.8	8.8E-03
cranberry	4	1	66	662	0.80	0.09	8.8	7.5E-08
dicyclomine	5	8	65	655	0.38	0.09	4.3	3.5E-04
ergocalciferol	3	5	67	658	0.38	0.09	4.1	6.8E-03
gabapentin	5	10	65	653	0.33	0.09	3.7	1.5E-03
hydrocortisone	7	17	63	646	0.29	0.09	3.3	8.8E-04
lacosamide	7	16	63	647	0.30	0.09	3.4	5.3E-04
leucovorin	3	5	67	658	0.38	0.09	4.1	6.8E-03
lorazepam	9	36	61	627	0.20	0.09	2.3	1.4E-02
lubiprostone	3	3	67	660	0.50	0.09	5.4	7.1E-04
metronidazole	4	3	66	660	0.57	0.09	6.3	1.7E-05
mupirocin	5	13	65	650	0.28	0.09	3.1	7.7E-03
naproxen	4	6	66	657	0.40	0.09	4.4	9.7E-04
nitrofurantoin	8	2	62	661	0.80	0.09	9.3	2.3E-14
polyethylene glycols	58	448	12	215	0.11	0.05	2.2	8.5E-03
ranitidine	13	60	57	603	0.18	0.09	2.1	1.1E-02
simethicone	11	43	59	620	0.20	0.09	2.3	4.9E-03
sulfamethoxazole / trimethoprim	3	2	67	661	0.60	0.09	6.5	1.2E-04
yeast	4	4	66	659	0.50	0.09	5.5	9.1E-05

Chi-squared test with Benjamini-Hochberg procedure FDR = 0.10. 733 individuals with Classic RTT, across 152 drugs taken by at least 5 individuals

By nature of its design the clinical history present within RSNHS is volunteered retrospectively and is subject to underreporting bias. A prospective study, directly assessing urologic symptoms of individuals with RTT compared against an age matched apparently healthy population would be necessary to provide a quantifiable estimate of the degree of increased risk for urological complications among individuals with RTT.

### Urological dysfunction is present in a mouse model of RTT

**Fig 1:**
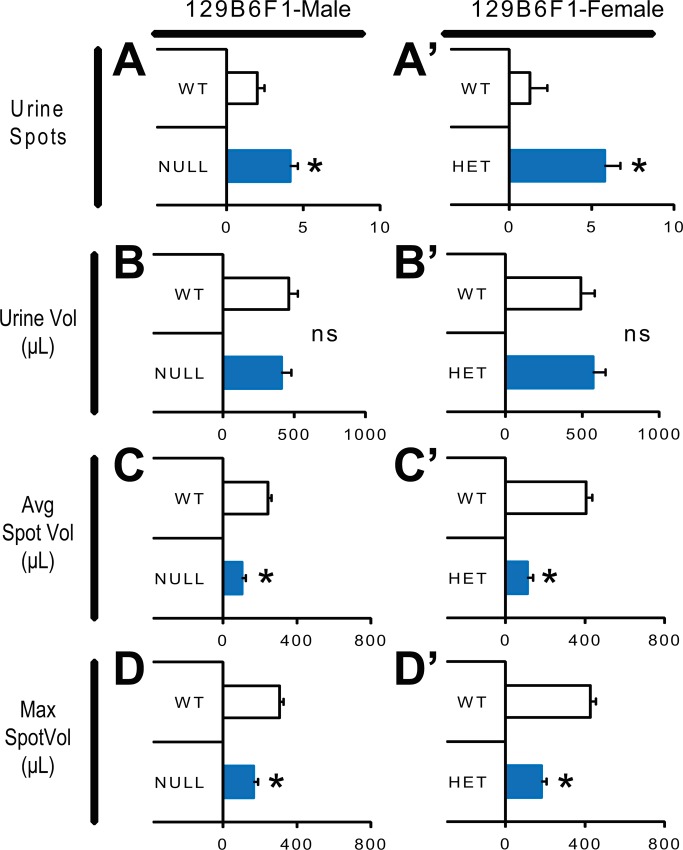
Micturition is impaired by loss of MeCP2 function. (A,A’) male *Mecp2*^*tm1*.*1Bird/Y*^ (NULL) and female *Mecp2*^*tm1*.*1Bird/+*^ (HET) mice showed abnormal micturition patterns with an increase in the number of micturition bouts, (B,B’) despite similar total volume of urine expelled during the test interval. (C,C’) Thus, the average volume voided per bout was decreased in NULL and HET mice relative to their WT littermates. (D,D’) Furthermore the maximum volume voided during the session was also reduced in the NULL and HET mice relative to WT littermates. Male 129B6F1 WT N = 8, NULL = 7; Female 129B6F1 WT = 5, HET = 6. *p<0.05 ANOVA for effect of genotype. ns not significant.

Because we observed evidence of urological dysfunction in people with RTT, we elected to assess whether loss of MeCP2 causes urologic dysfunction using a commonly used mouse model of RTT, mice possessing the *Mecp2*^*tm1*.*1Bird*^ allele. Both hemizygous male and heterozygous female mice with this allele have been extensively characterized with respect to *Mecp2* dependent effects on behavior, physiology, and survival [11, 20, 27, 28]. We characterized bladder function on an isogenic 129B6F1 strain background using male *Mecp2*^*tm1*.*1Bird/Y*^ (NULL) and female *Mecp2*^*tm1*.*1Bird/+*^ (HET) mice alongside gender matched wild type littermates (WT). We used Void Stain on Paper (VSOP) analysis to assess patterns of micturition. NULL mice on the 129B6F1 strain background showed an abnormal micturition pattern with an increased number of urine spots per VSOP session, and a decreased average volume of urine per spot as well as decreased maximum volume urinated at any spot (**Fig 1A–1D**). Because RTT is primarily diagnosed in females we also tested female mice to increase the generalizability of the results. Female 129B6F1 HET mice showed the same abnormal micturition pattern observed in the NULL mice (**Fig 1A’–1D’**), indicating that the micturition abnormality is not a gender specific effect.

**Fig 2:**
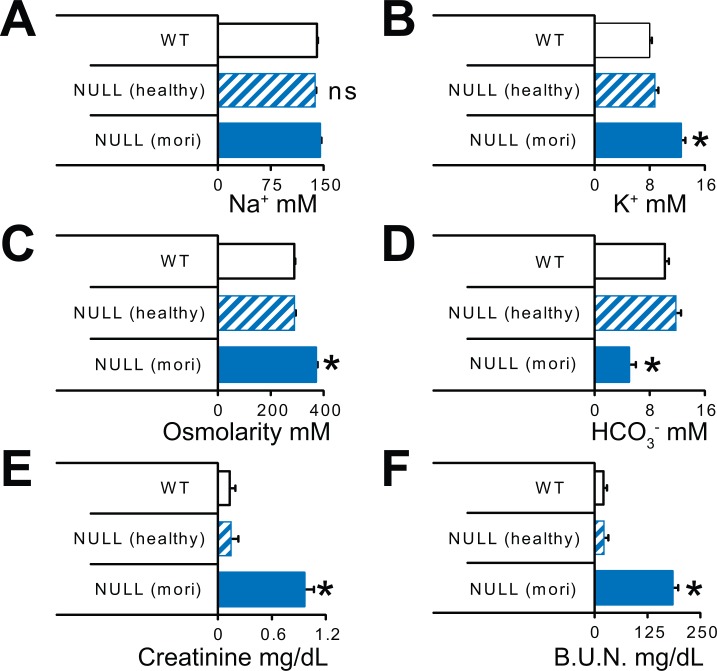
Loss of MeCP2 function contributes to kidney failure in male *Mecp2*^*tm1*.*1Bird/Y*^ mice. Moribund NULL mice (mori) exhibited evidence of kidney failure compared to normal serum chemistry values in WT or healthy NULL littermates before they become moribund. Moribund NULL mice exhibited (A) normal Na^+^, (B) increased K^+^, (C) increased serum osmolarity, (D) decreased bicarbonate, (E) increased creatinine, and (F) increased blood urea nitrogen levels. (WT N≥17, NULL healthy N≥9, NULL moribund N≥7). *p<0.05 versus all other groups determined by ANOVA with Bonferroni *post hoc* correction for multiple comparisons.

Abnormalities in micturition can ultimately lead to urine reflux from the bladder into the kidneys and cause kidney damage. To determine if kidney function was affected in animals lacking MeCP2 function, we assessed blood serum markers of kidney function in male WT, healthy NULL, and moribund NULL mice raised on a 129B6F1 strain background. Moribund NULL mice exhibited several serum markers of impaired kidney function, with increases in potassium, creatinine, blood urea nitrogen, and osmolarity, along with a sharp drop in bicarbonate levels (**Fig 2**).

### Kidney failure is a cause of mortality in male mice lacking MeCP2

**Fig 3:**
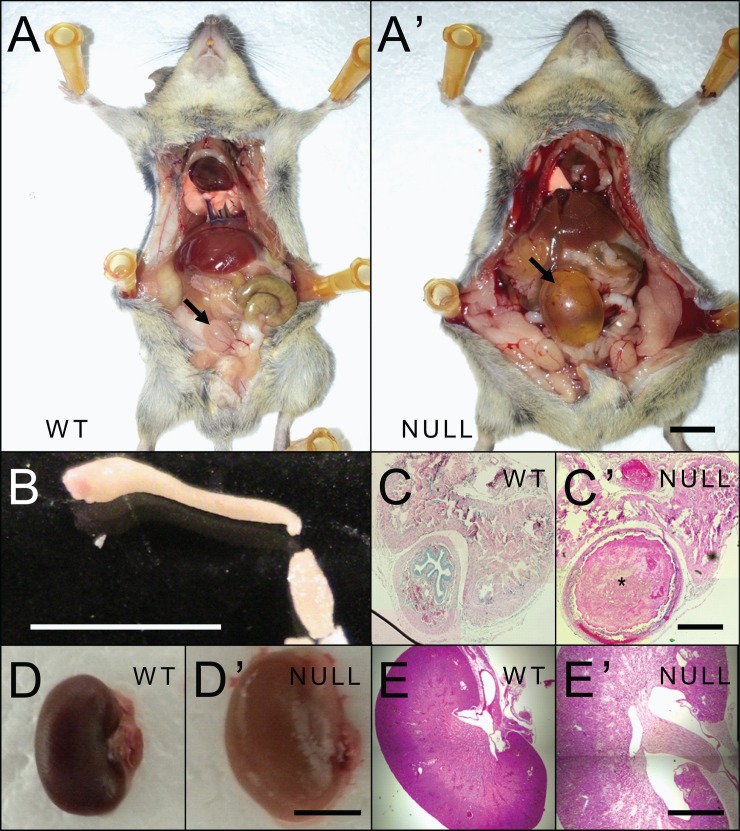
Representative urological pathology of 129B6F1 *Mecp2*^*tm1*.*1Bird/Y*^ mice. (A-A’) Gross examination of moribund NULL mice revealed severely distended bladders compared to WT littermates. Bladders indicated by black arrows. (B) A plug of seminal coagulum was present within the penile urethra of the moribund NULL mice. (C-C’) Coronal H&E stained sections through the penile urethra showed that this plug occludes the urethra, effectively limiting urine outflow. Occlusion of the urethra is indicated by * in C’. (D-E’) The kidneys exhibited moderate hydronephrosis observable at both the gross level (D-D’), and in coronal H&E stained sections taken through the middle of the kidney (E-E’). Scale bars: A-A’ 1cm, B 5mm, C-C’ 250μm, D-D’ 5mm, E-E’ 2mm.

We decided to examine the moribund NULL mice by necropsy to determine if there were any gross pathology or histological explanations for the mice to be in kidney failure. We identified severely distended bladders in the moribund male NULL mice raised on a 129B6F1 background. Further dissection and histology through the urogenital system revealed that the penile urethras of the NULL mice were obstructed and unable to pass urine, resulting in moderate hydronephrosis visible upon gross dissection of the kidneys and within H&E stained sections taken through the midline of the kidney (**Fig 3**). Histological staining of the obstruction was consistent with seminal coagulum as the source material.

Given the abnormal cause of death we next sought to determine the extent to which the phenomenon was specific to particular *Mecp2* alleles or mouse strain backgrounds. To determine whether this phenotype is dependent on specific *Mecp2* alleles, we generated *Mecp2*^*tm1*.*1Jae/Y*^ and *Mecp2*^*tm2Bird/Y*^ mice on a 129B6F1 strain background. In both cases, all mutant mice (n = 3 for each mutant allele) developed obstructed urethra and distended bladder pathology indicating that loss of function of MeCP2 on a 129B6F1 background will lead to urological dysfunction regardless of *Mecp2* allele.

**Fig 4:**
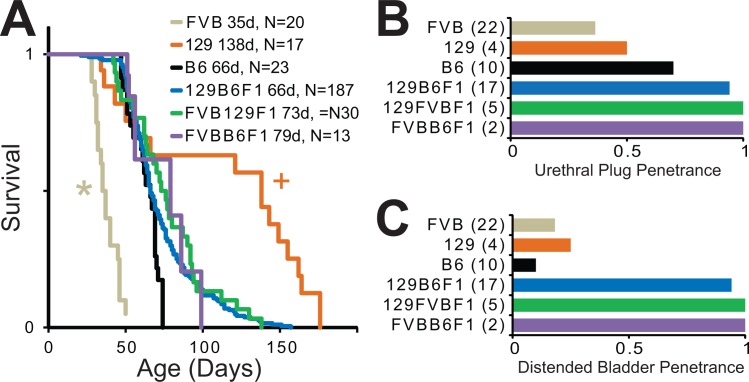
Strain dependent survival in *Mecp2*^*tm1*.*1Bird/Y*^ mice and penetrance of urethral obstruction. (A) Survival plots of male *Mecp2*^*tm1*.*1Bird/Y*^ (NULL) mice show premature lethality across all strains tested. FVB NULL mice died significantly earlier than all strains and 129 NULL mice lived significantly longer than 129B6F1 mice. *p<0.05 versus all other strains, and +p<0.05 versus 129B6F1 and FVB as determined by Tarone-Ware statistics from Kaplan-Meier analysis following Bonferroni *post hoc* correction for multiple comparisons (15 comparisons). (B) Penetrance of urethral obstruction in male moribund NULL mice varied by strain (p<0.001 determined by Chi-Square) with pure strains showing lower penetrance than isogenic F1 strains. (C) Similarly, penetrance of distended bladder while moribund also varied by strain (p<0.001 determined by Chi-Square) and was lower in the pure strains and highest in the isogenic F1 strains. N for each strain is indicated on the respective graphs. Median survival in days is indicated in the key for panel A.

To address the issue of strain, we characterized the deaths of male NULL mice maintained on congenic 129S6, C57BL/6, and FVBHSD strains, as well as isogenic F1 strain combinations. Interestingly, most of the strains possessed similar survival curves with the exception of the mice on the congenic FVBHSD strain background that had earlier death, and mice on the congenic 129S6 background in which a subset of animals live much longer than the other strains (**Fig 4**). Furthermore, penetrance of the urethral obstruction and distended bladders varied across the tested strain backgrounds, with lower penetrance on pure congenic backgrounds and nearly 100% penetrance on isogenic F1 backgrounds. Interestingly, the penetrance of these phenotypes showed no direct relationship to the duration of survival of the animals, with both the longest- and shortest-lived strains among the groups with the lowest penetrance of urethral plug and distended bladder phenotypes. This may indicate that these short- or long-lived strains actually die of a different cause.

## Discussion

In this work we identified evidence of urologic complications in a large sample of individuals with RTT and coincidental demonstration of urological dysfunction in both male and female mouse models of RTT. These urological abnormalities have yet to be clearly delineated in RTT but may represent a significant clinical issue impacting both quality of life and risk for serious medical complications. Because of this, a prospective study investigating urological function among individuals with RTT is needed to quantitatively assess risk of urological complications impacting overall health and quality of life.

The overall occurrence of urological pathology in individuals with mutations in *MECP2* was similar across typical, atypical and non-RTT diagnoses suggesting that the problems may be more generally related to neurological impairments arising from aberrant *MECP2* function. The more common urological complications observed in neurodevelopment disorders are urgency and incontinence, or problems with persistent urine retention [12, 15, 16].

The RSNHS data analyzed contained 8 years of natural history data from individuals with RTT represents 9320 person-years of data. This suggests that the 3 most common reported urological problems among individuals with RTT have annual incidence rates of 5/1000 individuals developing UTI, 3.4/1000 developing kidney stones, and 1.2/1000 developing urine retention. The incidence rate of UTI among the general population of females up to age 6 seems to be higher with pediatric cases reported at 7-14/1000 individuals [29, 30]. However, the rates of kidney stones and urinary retention are higher among individuals with RTT, with the general population developing kidney stones at an annual rate of 0.5-1/1000 individuals, and urinary retention in the general population of females occurring at an annual rate of 0.07/1000 individuals [31–33].

Urologic complications have been described in disorders with mobility impairments such as Cerebral Palsy. Some estimates of UTI in patients with Cerebral Palsy suggest rates of occurrence 10 fold greater than the general population [34]. Furthermore, among a small sample of Cerebral Palsy patients referred for urologic symptoms 20 of 27 had incontinence, 2 of 27 had retention problems, and 13 of 27 had a history of UTI [35]. A systematic meta-analysis of 27 studies on lower urinary tract symptoms in individuals with Cerebral Palsy suggest more than half develop lower urinary tract symptoms at some point [36]. It is difficult to make direct comparisons on prevalence and incidence rates between these studies on individuals with Cerebral Palsy with our results on individuals with RTT. However, it is possible that shared features, such as mobility deficits, may be a common source of urologic complications between RTT, Cerebral Palsy, and other neurological disorders.

Kidney stones are not commonly observed in rodent models and are typically only seen in genetic models of increased stone formation that are also subjected to additional exacerbating treatments [37]. However, lower urinary tract symptoms have been observed by investigating micturition patterns in mice and may reflect retention, or incontinence [38]. In the process of examining a mouse model of RTT for abnormalities in micturition output, kidney function, and urological histopathology, we identified urological dysfunction in both male NULL mice and female HET mice. Mice lacking MeCP2 function display abnormal patterns of micturition, with increased bouts of micturition and decreased volumes voided per bout. These findings are consistent with our human data and suggest that MeCP2 may play a role in urinary tract function.

Unexpectedly, we also discovered that one potential cause of early death in male NULL mice was kidney failure due to bladder dysfunction. Similar obstructive uropathies have been identified in other mouse models [39–41]. It should be noted that the described urethral plugs differ from those previously reported to occur within the proximal urethra near the bladders of healthy laboratory rodent species which may promote retention of urine without causing complete obstruction of urinary outflow[42]. The urethral plugs in the male RTT mice are found in the penile urethras and are able to completely obstruct urine outflow. There are several mouse lines that exhibit similar lethal urethral obstructions with varying frequencies under normal living conditions, or are prone to induction of urethral plug formation leading to obstructive uropathy [39–41, 43–46].

In a breeding population of WT C57BL/6N mice, 8.5% of males generated a urethral plug in the distal urethra resulting in bladder distension and irritation that increased rates of penile mutilation [44]. Additionally, the diabetic model KK-A^y^ is reported to have approximately 75% penetrance of urethral plugs leading to obstructive kidney failure, representing the largest contributor to spontaneous death observed in these animals [41]. Interestingly, the penetrance of the obstructive uropathy pathology in the KK-A^y^ mice can be modified by dietary intervention suggesting it may result from complications of the metabolic abnormalities in the mice. The idiopathic epilepsy EL mouse line also exhibits obstructive uropathy due to seminal coagulum plug blocking the distal urethra. The EL mice have 94% penetrance of this phenotype by 300 days of age, with the obstructions occurring after the mice have reached sexual maturity which is also coincidental with the typical age of onset for seizures in these mice. Additionally, administration of a ketogenic diet which prevents seizures in the EL mice also prevents the occurrence of the obstructive uropathy, however previous linkage analysis failed to find correlation of the uropathy with inheritance of known epilepsy susceptibility loci from the EL mice [40]. Also, treatment of C57BL/6J mice with an anesthetic cocktail of medetomidine and ketamine causes obstructive urethral plugs in 3% of male mice, possibly as a result of spontaneous ejaculation caused by the drugs [39].

Across these studies, the likely etiology of the obstructive urethral plugs ranges from predisposing urethral restrictions, insufficient bladder motility limiting the ability of urine to clear the urethra, and inappropriate spontaneous or incomplete ejaculation that allows the ejaculate to coagulate along the distal urethra. Thus, for the male NULL mice, the suggested etiology of the urethral obstructions involve the mice retaining ejaculate in the urethra that then coagulates and accumulates becoming impacted at the bulbourethral gland until urine outflow is inhibited. The impaired urine outflow then contributes to the development of mild hydronephrosis and impaired kidney function resulting in death. It will be interesting to determine if the micturition phenotype correlates with the penetrance of the obstructive uropathy on other strains of NULL mice as suggested by the 129B6F1 strain on which it was originally observed.

Understanding the causes of mortality in animal models of disease is important for the correct interpretations of data based on differential survival, and provides a framework to determine the degree to which translation of survival modifying treatments are mechanistically valid for human patients. The premature lethality of the male NULL mice made them an attractive model to test hypotheses regarding causes of and treatments for sudden unexpected death that occur in a subset of RTT patients [47–49]. However, this was based on an assumption that the cause of lethality in the male NULL mice was translatable to the human condition. Given that the incidence of this peculiar phenotype is specific to the male anatomy, its relation to mortality in females with RTT is unlikely. This raises the concern that treatments and genetic modifiers evaluated primarily on survival with little focus on other behavioral or physiological deficits may simply identify modulators of the onset of obstructive kidney failure.

The decreased incidence of obstructive uropathy on the FVBHSD strain was likely due to the early age of death observed in mice lacking MeCP2 function on this genetic strain background. Specifically, most mice die before reaching sexual maturity and may simply be less likely to produce the ejaculate required to form the urethral plug. Use of strains with lower penetrance of this phenomenon, such as C57BL/6J or FVBHSD, is recommended to prevent negative findings for pre-clinical studies that make use of survival as a metric of efficacy. Furthermore, these findings underscore the need for additional metrics other than survival, and validation of observed effects in female HET mice. This recommendation is based on the potential for treatments to address other potential causes of death without modifying the onset of urethral obstruction or vice versa. Furthermore, identification of critical cellular populations by genetic conditional knock out experiments should be performed with complimentary conditional rescue experiments as the anatomic origin of the urethral obstruction may differ from that of other potential causes of death.

## Supporting Information

S1 File Human data from the Rett Syndrome Natural History Study used for the current study. Medications: data indicating the occurrence of urological dysfunction in patients that had also ever taken the listed medications or treatments. Css mba urologic symptoms: data indicating the occurrence of urological dysfunction in patients and their sub scores for the Clinical Severity Score and Motor Behavior Assessment questionnaires.
(XLSX)

S2 File Mouse data across the assays and reported parameters used for the current study. VSOP: data from the void stain on paper test for bladder function in mice used in the current study. Serum chem: data indicating serum chemistry values from mice used in the current study. Penetrance: data indicating the penetrance of urethral obstruction and distended bladder in mice used in the current study. Survival Data: data indicating the age and survival status of mice used in the current study.
(XLSX)
